# The Improvement of Nonspecific Chronic Symptoms Post-Gallbladder Clip Removal

**DOI:** 10.7759/cureus.30625

**Published:** 2022-10-24

**Authors:** Tarek Abi El Cheikh, Iris Monica Vargas, Era Alili, Frederick Tiesenga

**Affiliations:** 1 Surgery, Washington University of Health and Science, Chicago, USA; 2 Surgery, Saint James School of Medicine, Chicago, USA; 3 Surgery, St. George’s University, Chicago, USA; 4 General Surgery, West Suburban Medical Center, Chicago, USA

**Keywords:** gallbladder clips, allergic reactions, metal allergies, nickel allergies, laparoscopic cholecystectomy, hypersensitivity reactions

## Abstract

Laparoscopic cholecystectomy is the first line of treatment for patients with symptomatic gallstones. Surgical clips made of stainless steel are still used by surgeons during these laparoscopic procedures and are considered relatively safe. These surgical clips, however, have been linked to severe side effects, including weight loss, dermatitis, angioedema, sleep hyperhidrosis, and more. This report documents the case of a 71-year-old female who presented for the removal of surgical stainless steel clips post-cholecystectomy after 16 years of chronic manifestations, including rashes on the torso and breasts, hives, weight loss, chills, and neuropathy due to a previously undiagnosed nickel allergy.

## Introduction

Laparoscopic cholecystectomy is one of the most performed procedures in the United States, partly because gallstone disease remains a prevalent condition. Over 20-25 million Americans are affected by gallstone disease yearly [1,2]. Laparoscopic cholecystectomy accounts for 90% of all cholecystectomies. Its reduced cost and potential for decreasing the length of hospital stay for a patient have contributed to making it the standard of care [3,4]. Surgical clips are commonly applied intraoperatively, as an alternative to ligation, to occlude the cystic duct and artery to prevent leaking and bleeding [5]. These clips, however, can sometimes dislodge and lie within the abdominal cavity following laparoscopic cholecystectomy [6,7].

More recently, various cases have been reported documenting the occurrence of allergies to nickel, titanium, cobalt, and chromium caused by surgical clips following laparoscopic cholecystectomy [8-12]. Surgical clips are commonly made of one of the following three alloys: titanium alloy (90% titanium, 5%-7% aluminum, 3%-5% vanadium, and <0.02% nickel), stainless steel (40%-68% iron, 8%-35% nickel, 20% chromium, 2% manganese, and 2%-3% molybdenum), and cobalt-chromium-molybdenum (60% cobalt, 27%-30% chromium, and <5% nickel) [9].

Because of its intrinsic and practical characteristics such as resistance to high temperatures and deterioration, good tensile strength, and low manufacturing cost, nickel has been commonly used in surgical tools such as clips, forceps, clamps, scissors, holders, and implants [13]. However, one of nickel’s most significant drawbacks is the prevalence of allergic reactions to it among the general population. Approximately 17% of females and 3% of males are afflicted with nickel allergy [14]. Contact dermatitis in type IV hypersensitivity reaction, lichen planus, dyshidrotic eczema, and burning mouth syndrome are some syndromes that can present because of nickel allergy [13]. Physical findings during the acute phase of the reaction can include erythema, induration, vesicles, bullae, scaling plaques, and edema. Findings during the chronic reactions include dryness, hyperkeratosis, pruritus, scaling, lichenification, hyperpigmentation, and fissuring [15].

## Case presentation

This case involves a 71-year-old female who presented with nonspecific symptoms involving multiple body systems due to nickel allergy for several years. The patient’s past medical history is significant for gastritis, primary hyperthyroidism, marginal zone lymphoma, cataracts of both eyes, vertigo, bilateral leg weakness, hip pain, neuropathy, and osteopenia. Her past surgical history includes laparoscopic cholecystectomy in 2006, total hip arthroplasty in 2016, and parathyroidectomy in 2019. The patient is allergic to latex, gold, copper, manganese, and nickel, a fact that remained unknown until she underwent testing in 2021 using memory lymphocyte immunostimulation assay (MELISA). She is currently on albuterol 90 mcg, two puffs every six hours, Allegra one tablet daily, vitamin D3 1,000-unit one tablet daily, diazepam 5 mg half tablet as needed, and gabapentin 300 mg three times a day.

In June of 2021, an autoimmune workup came negative for Sjogren’s and systemic lupus erythematosus, inflammatory arthropathy, vasculitis, autoinflammatory syndrome, and sarcoidosis. In July of 2021, initial metal patch testing did not show sensitivity to common metals but did show sensitivity to chlorhexidine. She then underwent a lymphocyte transformation test, which showed sensitivity to nickel, and it was confirmed afterward that she is allergic to nickel by MELISA. Around that time, she began a low-nickel diet, improving her general symptoms. In August of 2021, repeat allergy testing came positive for palladium chloride, vanadium, and trichloride. In addition, a neurodiagnostic skin biopsy was performed to rule out small fiber neuropathy, which was negative. The myositis panel also came back negative.

The patient had ongoing widespread symptoms, mainly fatigue, neuropathy, and a widespread rash, significantly impacting her function. She had improvement with symptoms on a nickel-free diet and Allegra after she learned that the clips used in her gallbladder surgery were made of stainless steel and contained nickel. Considering the high possibility that the nickel surgical clips placed during her cholecystectomy might be the cause of her symptoms, the patient decided to pursue the removal of the surgical clips after discussing it with her primary physician.

**Figure 1:**
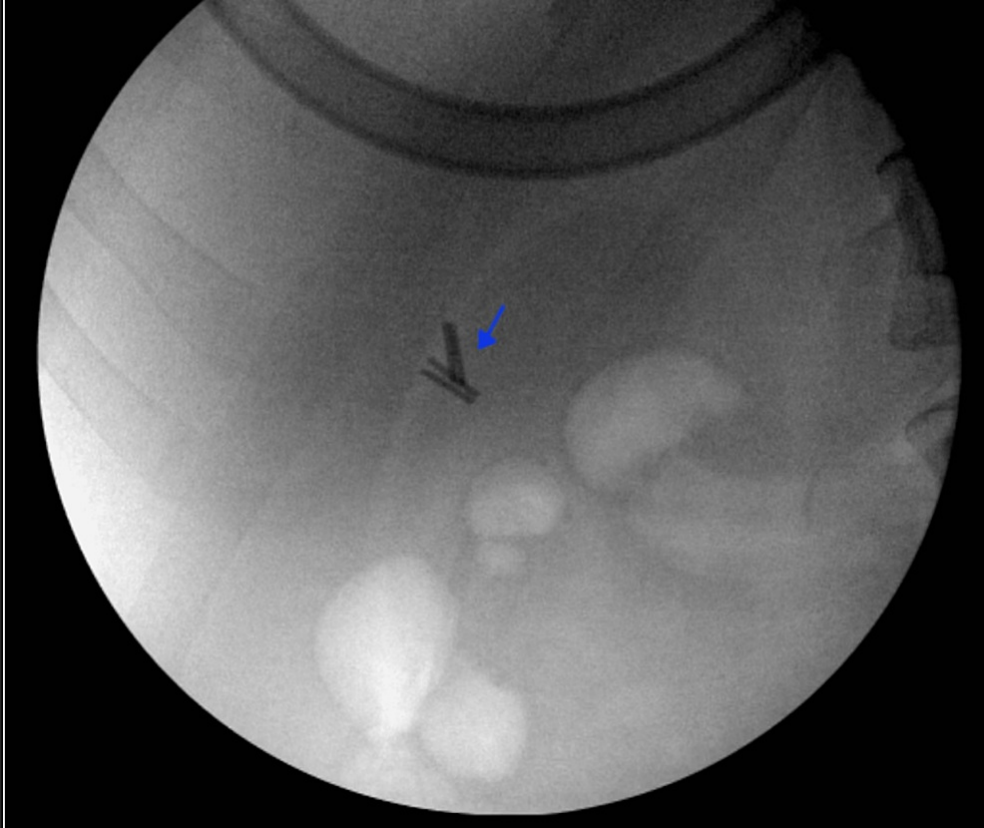
Diagnostic laparoscopy evaluation with fluoroscopic guidance located the positioning of four metal surgical clips (blue arrow).

**Figure 2:**
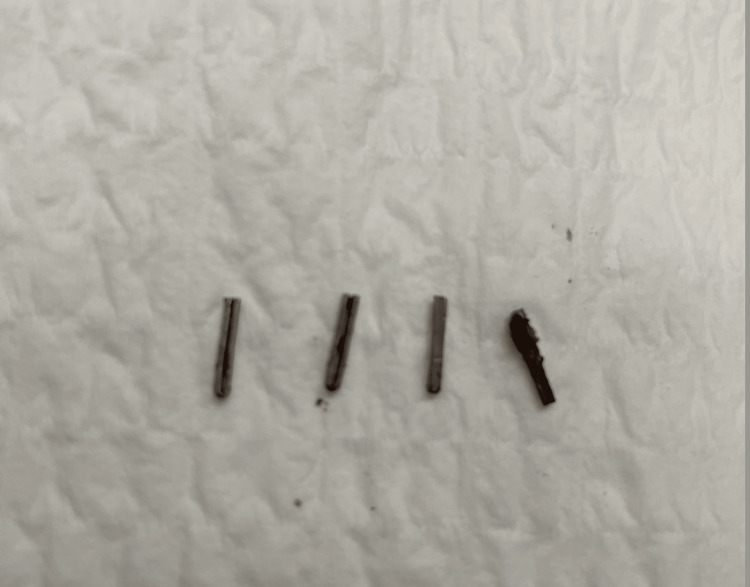
Four stainless steel surgical clips were removed from the right upper quadrant.

After consent was obtained, the patient was taken to the operative suite, where she was prepped and draped after satisfactory induction of general anesthesia. The abdomen was explored, and then, epigastric, mid-epigastric, and right upper quadrant ports were placed percutaneously under direct vision. Fluoroscopy showed four clips in the gallbladder fossa (Figure 1). The gallbladder fossa was then approached, adhesions were taken down with cautery, and four separate clips were identified and removed without event (Figure 2). Repeat fluoroscopy showed no evidence of retained clips. The surgical pathology report specified four gallbladder clips with no discernible tissue associated with the clips, and the clips are intact without abnormality.

The patient tolerated the procedure well, and there were no postoperative complications. She recovered well, and three months post-surgery, the patient reports improvement in all symptoms she experienced: neuropathy has improved, she is not taking gabapentin anymore, there were no more rashes, her fatigue significantly improved, and her weight is stable with no recent weight loss.

## Discussion

In this report, we presented the case of a patient who underwent a laparoscopic cholecystectomy at 55 years of age and two years later developed nonspecific symptoms. Symptom severity worsened until, at 71 years of age, the possibility of delayed hypersensitivity to the metal used in the surgical clips was considered. She underwent allergy testing, which confirmed the suspicion, 16 years after her cholecystectomy. Our study adds to a growing body of literature demonstrating the occurrence of metal-induced delayed hypersensitivity as a cause of systemic symptoms, even a decade or more after laparoscopic cholecystectomy [8,11,16] or other surgical procedures requiring metal implants [9,12,14].

In a previous case reported by our group, a patient presented with delayed hypersensitivity type IV symptoms that included abdominal pain, fatigue, lethargy, arthralgia, and nausea after cholecystectomy [8]. In the present case, our patient presented with rashes, mainly on the torso and breast, hives, night sweats, chills, and weight loss. In both cases, the patient’s symptoms persisted for more than a decade before they were diagnosed and first appeared at least two years after the laparoscopic cholecystectomy. In comparison, other case reports document initial symptoms presenting as early as five days after laparoscopic cholecystectomy [17] to more than a decade later in a spinal arthrodesis case [18]. Symptoms persisted for four weeks to 15 years [11] until they were correctly diagnosed and treated by surgical removal of the implant containing the offending metal.

T cells mediate type IV hypersensitivities by triggering inflammatory reactions in response to the presence of antigens, either exogenous or endogenous. An initial local immune and inflammatory response attracts leukocytes. Macrophages and monocytes engulf these antigens and present them to T cells. The latter become sensitized and activated, releasing cytokines and chemokines, which may cause tissue damage and illnesses [19].

Allergic reactions to antigens such as nickel, the metal present in the surgical clips removed from the patient presented in this report, are common in the general population. The prevalence of nickel allergy is approximately 17% in females and 3% in males [14]. Currently, preoperative testing for nickel allergy is not routinely performed, and patients are seldom aware of having these susceptibilities. Consequently, postoperative complications after patient exposure to implanted devices with nickel, such as clips or staples, may be more common than currently recognized, leading to chronic, systemic reactions that can be challenging to identify [11,15].

Post-surgery, symptoms resolved quite dramatically and led to a discontinuation of pain medication and the resolution of rashes that had been present for many years. The fact that the patient was placed on both a gluten-free diet and a nickel-free diet could be a confounding variable, but the dietary changes began months before the clip removal was performed, while the improvement of the symptoms was much more closely related to the removal of the nickel-containing surgical clips.

This case report reiterates that delayed hypersensitivity reactions to metals should be part of the differential diagnosis in postsurgical complications even decades after a surgical procedure. This may be especially relevant in the elderly, where the allergic disease may be masked by age-related physiological function decline [20]. Further studies on surgically related allergies in this sector of the population, and adults in general, should be conducted to predict better and understand the context of these conditions as they vary with age and to guide timely diagnosis and treatment.

## Conclusions

A possible metal allergy could be considered when a patient presents with nonspecific, possibly life-threatening complications, as seen with the patient in this case report. Potential allergens should be investigated before procedures involving surgical clips by obtaining detailed history with an emphasis on any allergy history and performing metal sensitivities and patch tests when necessary. In addition, hypoallergenic clips or alternatives to metal clips, such as plastic clips or suture ligation of cystic duct and artery, should be considered in patients undergoing gallbladder surgery with known reactivity to various allergens.
